# Omeprazole promotes carcinogenesis of fore-stomach in mice with co-stimulation of nitrosamine

**DOI:** 10.18632/oncotarget.19696

**Published:** 2017-07-31

**Authors:** Lei Huang, Dong-Jiang Qi, Wei He, A-Man Xu

**Affiliations:** ^1^ Department of General Surgery, The First Affiliated Hospital of Anhui Medical University, Hefei, China; ^2^ Department of General Surgery, The Fourth Affiliated Hospital of Anhui Medical University, Hefei, China

**Keywords:** proton pump inhibitor, MNNG, carcinogenesis, lysosomal enzyme, randomized study

## Abstract

**Objectives:**

To investigate if oral omeprazole application induces cancers of fore and glandular stomach in mice.

**Methods:**

A total of 66 eligible male mice were randomly divided into 6 groups, which were treated with control reagent, low (6 mg/kg) and high dose omeprazole (30 mg/kg), N-methyl-N’-nitro-N-nitrosoguanidine (MNNG, 100 mg/L water), and MNNG plus low and high dose omeprazole, respectively. After 24 weeks, concentrations of acid phosphatase (ACP) and N-acetyl-β-D-glucosaminidase(NAG) in serum and spleen was examined, and p21 and mTOR levels in stomach were detected.

**Results:**

The mouse spleen weight index was smaller in the omeprazole group than the control group, and in the MNNG plus omeprazole groups than the MNNG group. In the fore-stomach, more carcinomas were observed in the MNNG plus omeprazole groups than in the MNNG group. In the glandular stomach, there existed more atypical hyperplasia cases in the MNNG plus omeprazole groups than the MNNG-treated group, and one carcinoma was induced in the MNNG plus high dose omeprazole group. Omeprazole alone caused minor gastric pathological changes. Omeprazole treatment lowered both serum and spleen ACP and NAG levels in both the non-MNNG-treated and MNNG-treated subgroups. In fore-stomach, there existed decreased p21 and mTOR levels in the omeprazole-treated groups than in the control group, and in the MNNG plus omeprazole groups than the MNNG-treated group.

**Conclusion:**

Omeprazole promotes carcinogenesis of the mouse fore-stomach but not the glandular stomach following treatment with MNNG. Lysosomal hydrolase activity was inhibited and some cancer-associated proteins was dysregulated, which requires further explorations.

## INTRODUCTION

Gastric cancer (GC) is one of the most common and lethal digestive malignancies worldwide [1, 2]. Its incidence and mortality is highest in Eastern Asia, especially in China [3]. Although overall there is a decreasing trend in incidence, the world is witnessing an increasing number of adenocarcinomas of the esophagogastric junction (EGJ) in recent decades [4, 5]. Incidence rates of esophageal squamous cell carcinoma have also been increasing especially in some Asian areas [6]. Nitrosamine intake and chronic *Helicobacter pylori* (*Hp*) infection are the key risk factors for GC [7, 8]. Eradication of *Hp* using proton pump inhibitors (PPIs) especially omeprazole and antibiotics is one of the classical and effective primary preventive strategies [9].

PPIs are essential and commonly used for the treatment of various digestive diseases, such as gastroesophageal reflux disease and peptic ulcers [10, 11]. Since first applied clinically in 1989 in the US, PPIs significantly reduces the gastroesophageal surgery rate [12]. However, a marked number of PPI prescriptions are often inappropriate, potentially resulting in diverse adverse effects, including gastric parietal cell hyperplasia, gland cyst, hypergastrinemia, fundic gland polyp, *etc.*, with the underlying mechanism barely revealed [13, 14]. A recent study [15] showed dose-dependent inhibition of lysosomal enzyme activities by PPIs in cultured cells and mouse spleen. PPIs counteracted tumor immunotherapy in the mouse model, suggesting that PPI-associated adverse effects might be caused by systematically compromised immunity.

It remains obscure whether long-term application of PPIs promotes carcinogenesis of EGJ and stomach due to lack of basic and clinical evidence, especially with the co-stimulation of nitrosamine. Currently there are few experiments administering PPIs to experimental animals for a relatively long period. This study investigated the influence of long-term gavage of different doses of omeprazole, the most classical PPIs, with/without N-methyl-N’-nitro-N-nitrosoguanidine (MNNG), an N-nitroso-compound, on mouse mucosa, to reveal whether omeprazole promotes carcinogenesis of EGJ and stomach and to explore the potential underlying mechanism, which will provide crucial theoretical basis for rational clinical drug application.

## RESULTS

### Effect of omeprazole and/or MNNG on mouse general conditions and SWI

**Figure 1:**
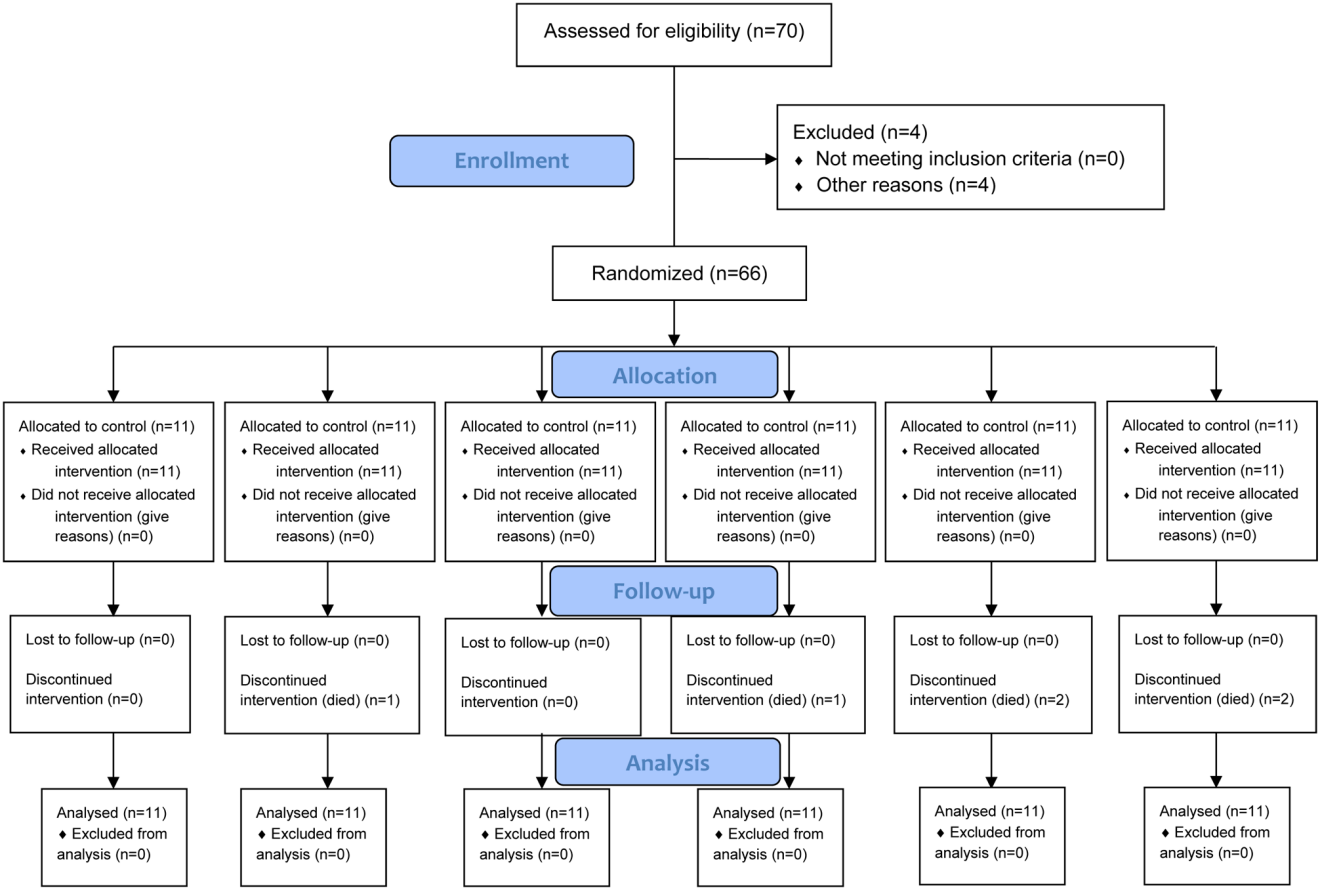
Flow diagram Based on intention-to-treat analysis.

**Figure 2:**
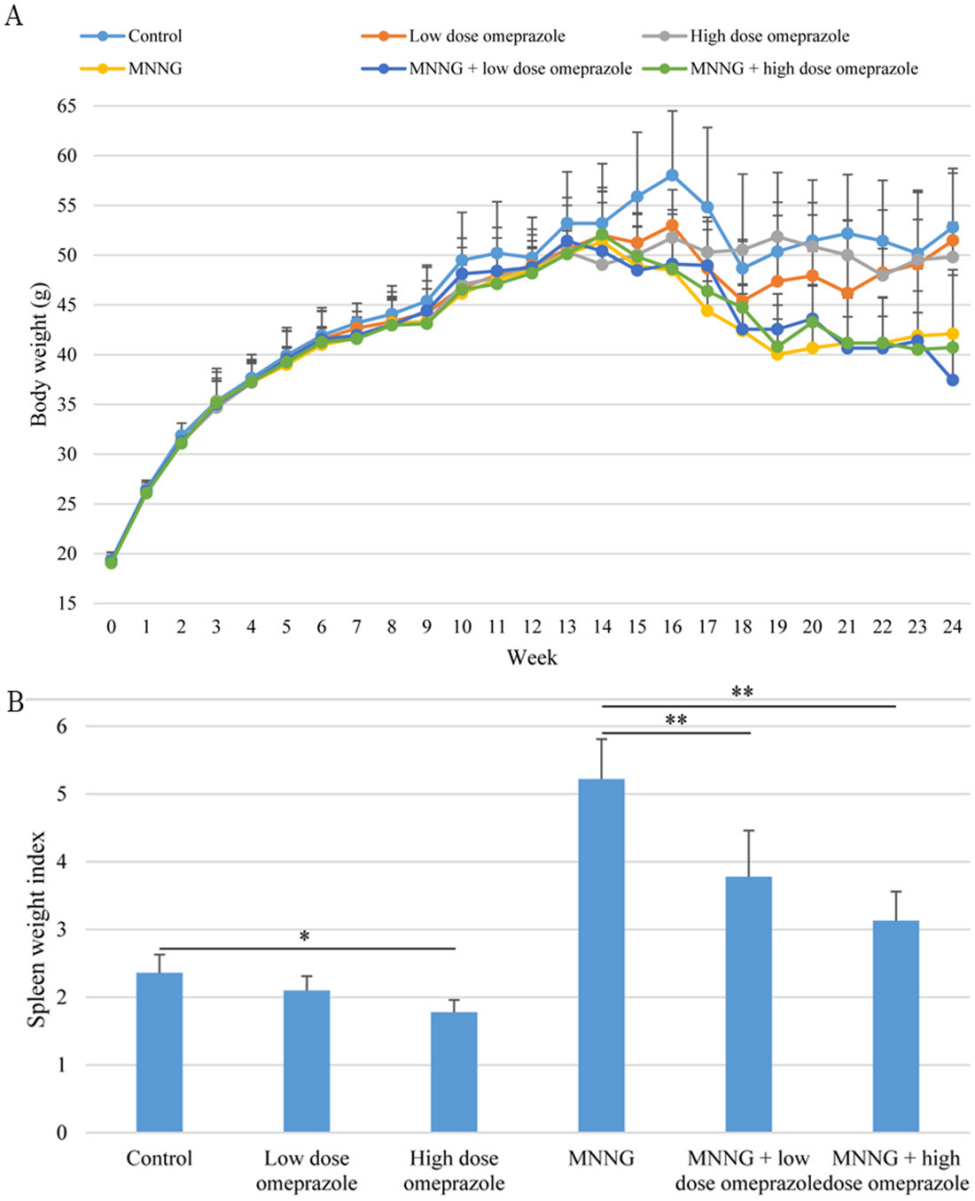
Body weight changes **(A)** and spleen weight index **(B).** The body weights in the MNNG-treated subgroups were lower than those in the non-MNNG-treated groups. Omeprazole treatment decreased the spleen weight index in both the non-MNNG-treated and MNNG-treated subgroups. MNNG, N-methyl-N’-nitro-N-nitrosoguanidine.

A total of 6 mice died during treatment (1 in the low dose omeprazole group, 1 in the MNNG-treated group, 2 in the MNNG + low dose omeprazole group, and 2 in the MNNG + high dose omeprazole group, Figure 1). This study was based on intention-to-treat analysis. The mice in the omeprazole-treated groups with/without MNNG supply were obviously more active, and had better hair color and increased food intake compared to the ones in the corresponding groups without omeprazole gavage. However, omeprazole treatment decreased mouse body weight, and mice were lighter in the MNNG-treated subgroup than in the non-MNNG-treated subgroup (Figure 2A).

The spleen weight (mg) and SWI were smaller in the low dose omeprazole group (128.8 ± 12.9 and 2.10 ± 0.21), and were significantly smaller in the high dose omeprazole group (104.0 ± 15.2 and 1.78 ± 0.18), when compared to the control group (93.0 ± 12.8 and 2.36 ± 0.27). They were significantly smaller in the MNNG plus low dose omeprazole group (138.0 ± 30.3 and 3.78 ± 0.68) and the MNNG plus high dose omeprazole group (115.0 ± 13.3 and 3.13 ± 0.43) when compared to the MNNG group (198.3 ± 35.2 and 5.22 ± 0.59). (Figure 2B).

### Pathological effect of omeprazole and/or MNNG on mouse stomach

**Figure 3:**
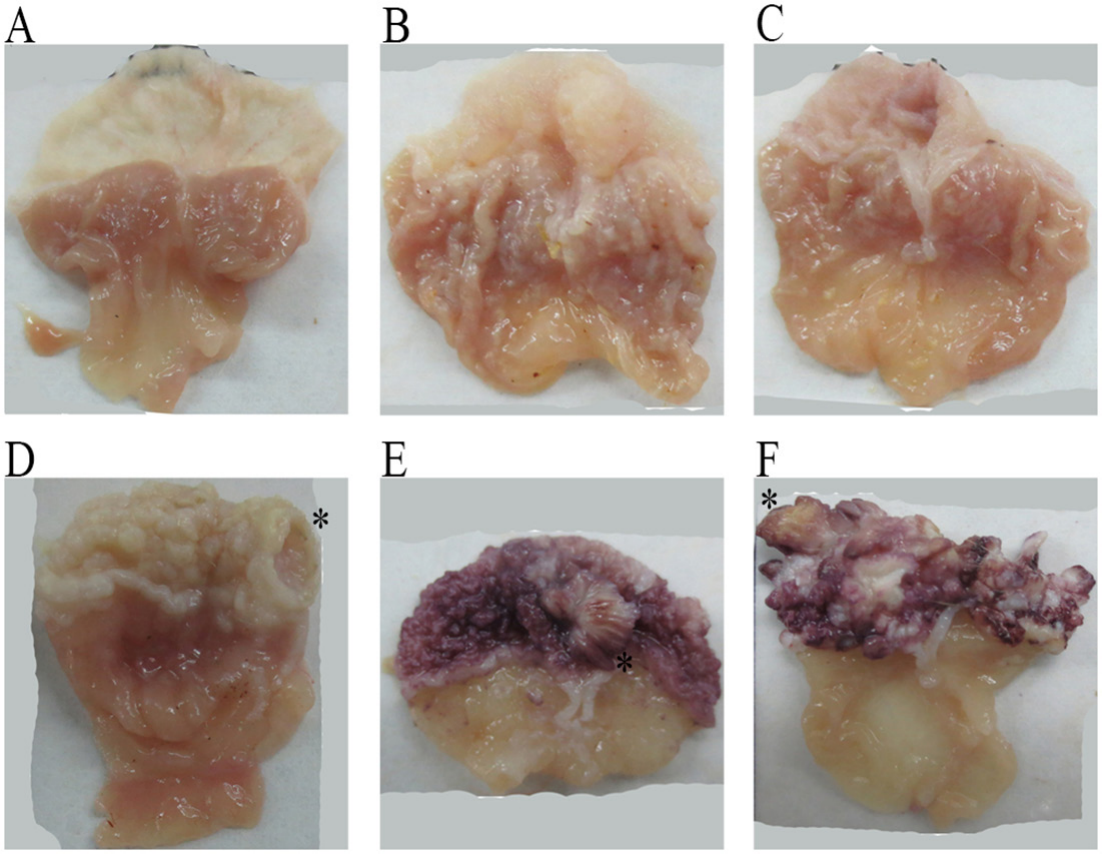
The gross mouse gastric specimens In the control group **(A)**, the mouse fore-head gastric mucosa was smooth, and the glandular stomach had intact mucosa, with obvious mucosal folds. In the omeprazole-treated groups **(B)**, low dose omeprazole; and **(C)**, high dose omeprazole), there existed papillary eminences in the fore-gastric mucosa, and the mucosal folds of the glandular mucosa were markedly shallower, especially in the high-dose group. In the MNNG-treated group **(D)**, ulcerative lesions occurred in the fore-stomach, with obvious mucosal hypertension, and the glandular gastric folds became shallower. In the groups treated by MNNG and omeprazole **(E)**, MNNG + low dose omeprazole; and **(F)**, MNNG + high dose omeprazole), there existed many eminent lesions in the fore-stomach, and the mucosal folds of the glandular stomach disappeared. The typical lesions in the omeprazole-treated groups were marked with ‘*’. MNNG, N-methyl-N’-nitro-N-nitrosoguanidine.

Macroscopically, the mouse gastric mucosae in the control group were smooth, with obvious mucosal folds in the glandular stomach, and there were no observable ulcer or eminence lesions. In the omeprazole groups, there were no significant changes in the fore-stomach, but the mucosal folds of the glandular stomach were shallower. In the 3 MNNG-treated groups, there existed many white papillary prominences of varying sizes on the fore-stomach mucosa, which were significantly thicker, with occasional ulcerative lesions. The mucosal folds of the glandular stomach were significantly shallower and close to disappearance, especially in the group treated with MNNG and high dose omeprazole. (Figure 3).

**Figure 4:**
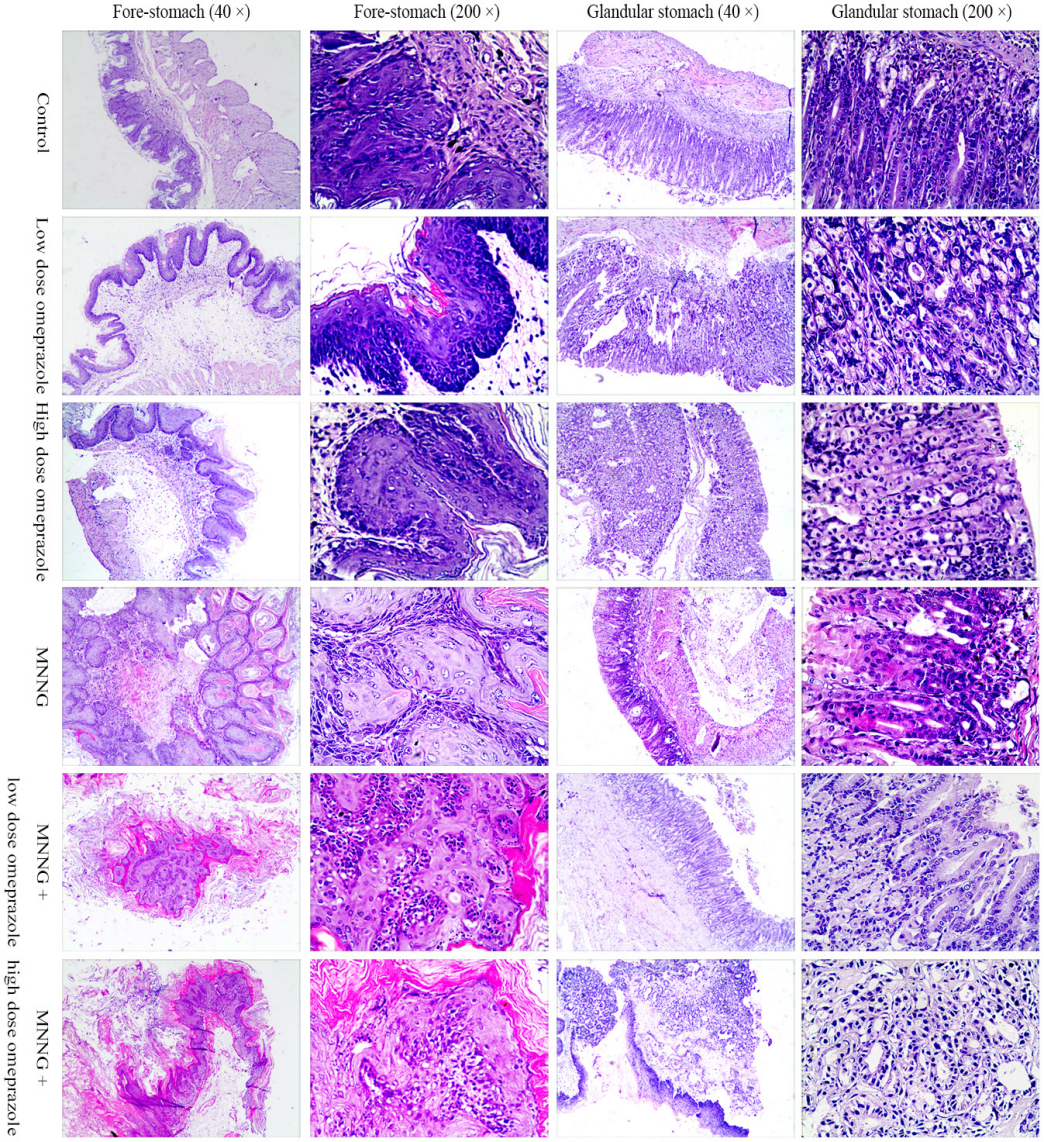
The pathology of mouse fore and glandular stomachs In the omeprazole-treated groups, the fore-stomach squamous epithelial cells underwent hyperkeratosis, and the basement cells were disorderly arranged, invading into the deeper layer in a papillary shape; in the glandular stomach, part of the glands presented atypical hyperplasia. The fore-stomach in the MNNG-treated groups presented a papillary proliferation status with hyperkeratosis and parakeratosis, and malignant cells were occasionally seen; however, the glandular stomach was basically normal. In the glandular stomach of the MNNG plus omeprazole groups, infiltration of inflammatory cells into the mucosal and sub-mucosal layers were prevalent, with more frequent glandular intestinal metaplasia, dysplasia and abnormal hyperplasia, some accompanied with adenocarcinomas. MNNG, N-methyl-N’-nitro-N-nitrosoguanidine.

After sacrifice, the mouse was dissected, and the stomach (including both the fore-stomach and glandular stomach) was obtained and sliced for microscopic observations. In the control group, the mouse fore-gastric mucosae were histologically normal, with clear structures of all layers and without inflammatory cell infiltration into the mucous or sub-mucosal layer. The gastric mucosa in the omeprazole-treated groups were histologically disorganized: in the fore-stomach, the squamous epithelial cells underwent hyperkeratosis, and the basement cells were disorderly arranged, invading into the deeper layer in a papillary shape; in the glandular stomach, part of the glands presented atypical hyperplasia, with inflammatory cells invading into the mucosal and sub-mucosal layers. The fore-stomach in the MNNG-treated groups presented a papillary proliferation status with hyperkeratosis and parakeratosis, and malignant cells were occasionally seen; however, the glandular stomach was more normal. In the MNNG plus omeprazole groups, infiltration of inflammatory cells into the mucosal and sub-mucosal layers of the glandular stomach were prevalent, with more frequent glandular intestinal metaplasia, dysplasia and abnormal hyperplasia, some accompanied with adenocarcinomas. (Figure 4) No metastatic lesions were detected for all the cancer cases.

### Omeprazole and/or MNNG-induced lesions in mouse stomach

In the fore-stomach, compared to the control group, there existed significantly more papillary hyperplasia cases in the low (45.5% *vs.* 0.0%, *P* = 0.035) and high (63.6% *vs.* 0.0%, *P* = 0.004) dose omeprazole-treated groups; no carcinomas were induced in the 3 groups. More carcinoma cases were observed in the MNNG plus low (36.4% *vs.* 9.1%, *P* = 0.117) and high (54.5% vs. 9.1%, *P* = 0.022) dose omeprazole groups than in the MNNG group, occasionally invading the deep muscular layer; papillary hyperplasia was induced in all MNNG-treated groups.

In the glandular stomach, no carcinoma or intestinal metaplasia cases were induced in the omeprazole-treated groups without MNNG; however, more atrophic gastritis and atypical hyperplasia cases were observed in the low (for both lesions, 9.1% *vs.* 0.0%, *P* = 0.306) and high (for both lesions, 18.2% *vs.* 0.0%, *P* = 0.138) dose omeprazole-treated groups compared to the controls. There existed more non-atrophic gastritis and atypical hyperplasia cases in the MNNG plus low (54.5% *vs.* 36.4%, *P* = 0.012; 36.4% *vs.* 9.1%, *P* = 0.127) and high (63.6% *vs.* 36.4%, *P* = 0.004; 36.4% *vs.* 9.1%, *P* = 0.127) dose omeprazole-treated groups than the MNNG-treated group; notably, one carcinoma was induced in the MNNG plus high does omeprazole group. (Table 1).

**Table 1:** The pathological changes of the mouse fore and glandular stomachs^a^

Group	*n*	Fore-stomach		Glandular stomach				
		Papillary hyperplasia	Carcinoma	Non-atrophic gastritis	Atrophic gastritis	Intestinal metaplasia	Atypical hyperplasia	Carcinoma
Control	11	0	0	0	0	0	0	0
Low dose omeprazole	11	5	0	0	1	0	1	0
High dose omeprazole	11	7	0	0	2	0	2	0
MNNG	11	7	1	4	0	0	1	0
MNNG + low dose omeprazole	11	7	4	6	0	0	4	0
MNNG + high dose omeprazole	11	7	6	7	0	1	4	1

^a^Based on intention-to-treat analysis.

### Influence of omeprazole and/or MNNG on mouse serum and spleen lysosomal enzymes

**Figure 5:**
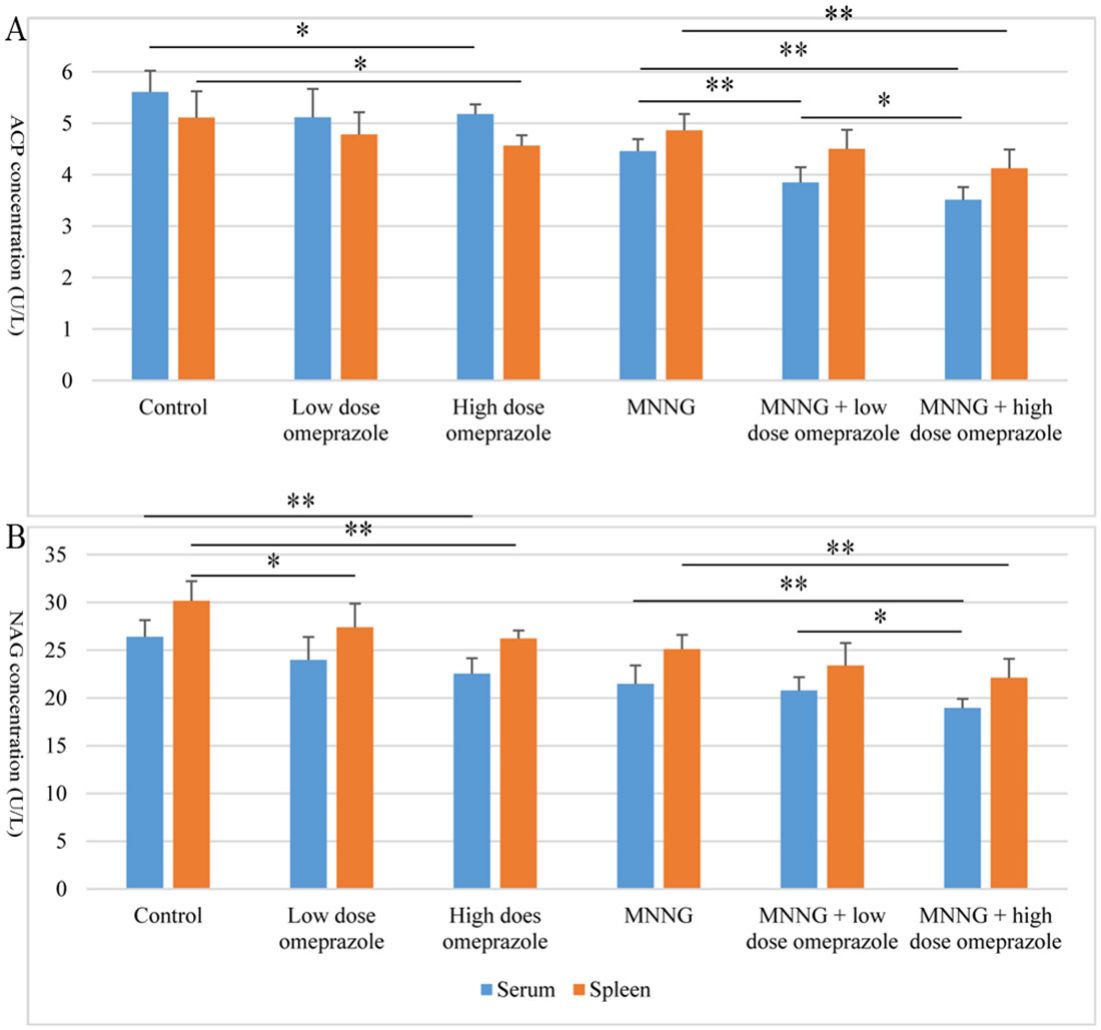
The ACP **(A)** and NAG **(B)** levels in mouse serum and spleen. In both the non-MNNG-treated and MNNG-treated subgroups, omeprazole treatment (especially at high dose) decreased both enzyme levels in both serum and spleen. ACP, acid phosphatase; NAG, N-acetyl-β-D-glucosaminidase; MNNG, N-methyl-N’-nitro-N-nitrosoguanidine. *, *P* < 0.05; **, *P* < 0.01.

The ACP levels were reduced after 24-week treatment of omeprazole in both the non-MNNG-treated and MNNG-treated subgroups. In serum, the ACP level was lower in both the high dose (*t* = -2.501, *P* = 0.028) and low dose (*t* = -1.892, *P* = 0.083) omeprazole groups than the control group; compared to the MNNG group, the ACP concentration was significantly lower in the MNNG plus omeprazole groups (MNNG + low dose omeprazole *vs.* MNNG: *t* = -4.315, *P* = 0.001; MNNG + high dose omeprazole *vs.* MNNG: *t* = -7.432, *P* = 0.000); and it was even lower in the MNNG + high dose omeprazole group compared to the MNNG + low dose omeprazole group (*t* = -2.326, *P* = 0.038). In spleen, the ACP level was lower in the high dose omeprazole group than the control group (*t* = -2.623, *P* = 0.031); compared to the MNNG group, the ACP concentration was lower in the MNNG plus omeprazole groups (MNNG + low dose omeprazole *vs.* MNNG: *t* = -1.960, *P* = 0.074; MNNG + high dose omeprazole *vs.* MNNG: *t* = -4.053, *P* = 0.002); and it was even lower in the MNNG + high dose omeprazole group compared to the MNNG + low dose omeprazole group (*t* = -1.926, *P* = 0.078). Interestingly, the ACP levels in the MNNG-treated subgroups were higher in spleen than in serum, which is opposed to the corresponding comparison patterns in the non-MNNG-treated subgroups. (Figure 5A).

The NAG levels were decreased after omeprazole treatment for 24 weeks in both the non-MNNG-treated and MNNG-treated subgroups. In serum, the NAG levels in the high dose (*t* = -4.309, *P* = 0.001) and low dose (*t* = -2.171, *P* = 0.051) omeprazole groups were both lower than those in the control group. The enzyme concentrations were lower in the MNNG plus high dose omeprazole group compared to the MNNG group (*t* = -3.111, *P* = 0.009) and the MNNG + low dose omeprazole group (*t* = -2.859, *P* = 0.014). In spleen, the NAG levels in the high dose (*t* = -4.716, *P* = 0.001) and low dose (*t* = -754, *P* = 0.042) omeprazole groups were both significantly lower than those in the control group. The enzyme concentration was lower in the MNNG plus high dose omeprazole group compared to the MNNG group (*t* = -3.211, *P* = 0.007). The NAG levels in both the non-MNNG-treated and MNNG-treated subgroups were higher in spleen than in serum. (Figure 5B).

### Expression of p21 and mTOR in mouse fore-stomach

**Figure 6:**
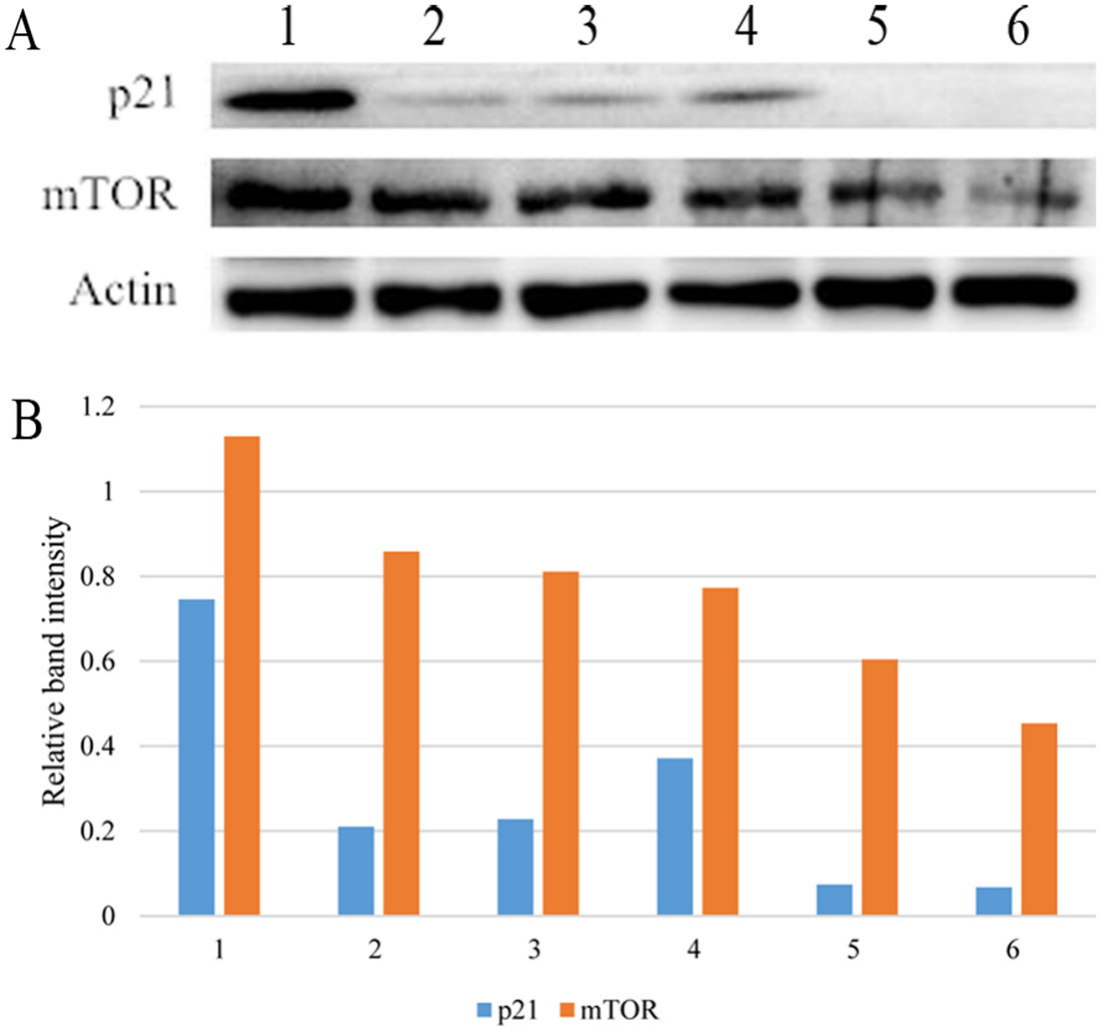
Western blotting **(A)** and relative band intensity to β-actin of p21 and mTOR **(B)**. Both proteins were down-regulated after omeprazole treatment in both the non-MNNG-treated and MNNG-treated subgroups. 1, control; 2, low dose omeprazole; 3, high dose omeprazole; 4, MNNG; 5, MNNG + low dose omeprazole; 6, MNNG + high dose omeprazole. MNNG, N-methyl-N’-nitro-N-nitrosoguanidine.

There were significantly decreased p21 and mTOR expression levels in the omeprazole-treated groups than in the control group, and in the MNNG plus omeprazole groups than in the MNNG-treated group. (Figure 6).

## DISCUSSION

Nowadays, PPIs especially omeprazole are widely overprescribed due to clinicians’ misinterpretation of their application indications. In Australia, Ireland, and England, respectively 63%, 33%, and 67% of patients receive PPI treatment inappropriately [13]. In the United States, about 69% of PPI prescriptions are lack of appropriate medical indications [16]. A recent study in China [17] revealed that there exists a gradually increased trend of the prevalence of PPIs-treated reflux diseases among adenocarcinoma of the GEJ, however, the mutual causality remains obscure. A report in the 1990s on effects of long-term omeprazole administration on chronic atrophic gastritis and phagocytic cell hyperplasia when comparing *Hp*-infected patients to *Hp*-negative cases evoked the concern that long-period PPIs usage might increase GC incidence [18]. In the past, the major concern regarding omeprazole use was the potential acceleration of gastric atrophy, due to migration of Hp proximally into the corpus [18]. A recent meta-analysis demonstrates that long-term PPI use is associated with increased risks of fundic gland polyps and GC [19]. The long-term application is thought to cause hypergastrinemia, which potentially increase the incidences of gastric and colorectal tumors [20, 21]. The acceleration of adenocarcinomas of the proximal stomach by omeprazole is primarily through its hypergastrinemia-inducing effect, which could be blocked by gastrin receptor antagonists. Besides, current evidence further supports that long-term PPIs application aggregates the *Hp*-induced chronic atrophic gastritis, intestinal metaplasia, and sequentially carcinoma [22, 23]. However, up till now, there is no evidence supporting that *Hp*-infected patients with a low acid secretion have an increased GC risk, and controversies remain on whether PPIs intake increases GC risks [20].

The two main types of esophageal cancer are squamous cell carcinoma and adenocarcinoma [1]. In high-risk areas, 90% of cases are squamous cell carcinomas, compared to about 25% in the US among white individuals [24, 25]. Contributing risk factors are not well understood in Asia, but are thought to include poor nutritional status, low intake of fruits and vegetables, drinking beverages at high temperatures, and potentially HPV infection [26–29]. In Western countries the primary risk factors for squamous cell carcinoma are alcohol and tobacco use, which account for almost 90% of total cases. The main known risk factors for esophageal adenocarcinoma are overweight and obesity and chronic gastroesophageal reflux disease (GERD), which can cause metaplastic changes to the esophagus (Barrett esophagus) predisposing to dysplasia and adenocarcinoma [30]. However, only a small percentage of those with Barrett esophagus go on to develop esophageal cancer [31]. Smoking and low intake of fruits and vegetables are also risk factors for adenocarcinoma of the esophagus. Notably, in high-risk areas the prevalence of established major risk factors for esophageal cancer is low [1].

In most parts of China, especially the rural and remote areas, people prefer pickled and smoked food, which contains a great amount of nitrosamine, which could be transformed into the highly carcinogenic N-nitroso compounds in stomach. Thus, the Asian pickled food has been categorized as a group 2B carcinogen. This study indicates that for such individuals, if omeprazole of relatively high-dose is applied for a long term, the incidence of adenocarcinoma of the proximal stomach might markedly increase. Thus, it is clinically significant in guiding the rational and standardized usage of PPIs.

Several studies have examined the effect of PPIs (*e.g.*, omeprazole) on gastric carcinogenesis, particularly in *Hp*-infected animal models. In mice, PPI administration increased the number of parietal cells with enhanced expression of water or ion transport proteins which were associated with fundic gland polyp development [32]. Acid suppression-induced hypergastrinemia promoted mucosal hyperplasia [33]. In *Hp*-infected Mongolian gerbils, long-term PPI administration was shown to promote adenocarcinoma genesis, which was associated with the progression of atrophic corpus gastritis [23]. Neuroendocrine tumor development was also enhanced in long-term high-dose PPI-treated gerbils [34]. Locally invasive neoplastic lesions prevailed in distal esophagus within PPI-treated rats [35]. Our study for the first time investigated the association between PPI administration and carcinogenesis at the present of a non-mouse-specific N-nitroso-compound.

This study showed that omeprazole treatment for a long term (especially at a high dose) significantly increased the tumor and dysplasia incidences of the mouse fore-stomach with the co-stimulation of MNNG, which is an N-nitroso-compound and a widely-existing active carcinogen, as confirmed by the pathological studies. However, no malignancy was induced by omeprazole alone. One of the potential reasons for the relatively low incidence of induced carcinomas might be the limited induction period. One of the tumorigenic mechanisms of PPIs is its causing hypergastrinemia via the inhibition of gastric acid production, which nourishes the epithelia especially the gastric mucosae, leading to their proliferation. However, the hypergastrinemia hypothesis could not explain the fact that the GC incidence is not significantly higher in patients with Zollinger-Ellison syndrome or receiving vagotomy. Notably, this study supported that relatively long-term application of PPIs alone did not remarkably induce cancer, but could significantly accelerate the carcinogenic process of high nitrosamine-intake animals. Besides, interestingly, omeprazole itself could increase the intra-gastric nitrosamine level, further reducing the vitamin C concentration, which is a potential risk factor for carcinoma [36]. We found that carcinomas were rarely induced in mouse glandular stomach. Some *in vitro* investigations even suggested that omeprazole might inhibit gastric antral carcinogenesis via suppressing proliferation and promoting apoptosis [37, 38]. Omeprazole might have only minor effects on promoting GCs. Unfortunately, the proliferation status was not specifically examined in our study, and further studies would be required in this regard.

This research further revealed that long-term omeprazole application significantly inhibited spleen and serum ACP and NAG, which are both typical lysosomal functional hydrolases, and which could reflect the overall enzyme level. It has been widely accepted that tumor initiation and development are closely associated with lysosome [39, 40]. The mitochondrial apoptotic and non-apoptotic programmed cell death pathways induced by the activation of lysosomal enzymes which are activated in acid environment could effectively kill tumor cells [41, 42]. The lysosomal homeostasis is disrupted in cancer cells, and the enzymatic ability markedly decreases during tumorigenesis [43]. PPIs could pass through the cell membrane freely in the lipophilic inactivated form, and lose this ability when protonated in acid environment. The fact that high acidity activates PPIs leads to the hypothesis that PPIs might potently inhibits lysosomes. PPIs are easily activated and prone to bind cysteine-containing peptides at pH of 5, and its systematic distribution potentially greatly inhibits the intracellular V-ATP enzyme and alkalizing the lysosome, which weakens its activities and facilitates cancer progression [15]. This study revealed that omeprazole treatment markedly inhibited both enzymes in mouse serum and spleen, indicating the deterioration of the lysosomal function. Besides, long-term omeprazole administration decreased mouse SWI, suggesting decreased systematic immune function, which potentially greatly promotes carcinogenesis.

Two key tumor-associated proteins p21 and mTOR were shown to be down-regulated after omeprazole treatment. The p21 protein is a negative regulator of the cell cycle. It halts the proliferative cells at a specific phase via inhibiting the corresponding cyclin-dependent kinases [44]. Its high expression indicates favorable prognosis in GC [45–47]. This study showed that omeprazole treatment could down-regulate the p21 expression in mouse stomach. Thus, omeprazole administration could worsen the outcomes of nitrosamine-induced GC. mTOR inhibits tumor proliferation and growth, induces apoptosis, and reverses drug-resistance [48]. Esomeprazole could inhibit the mTOR signaling pathway in melanoma, thus promoting tumor progression [49]. Consistently, this study showed that omeprazole inhibited mTOR expression during GC genesis. The MNNG intake will generate many reactive oxygen species, which further damages the DNA. The omeprazole supplied will inhibit the lysosomal function, impeding the repair and recycle of the accumulating unstable genome, and finally inducing cancer.

This investigation has some limitations. Firstly, gastrin levels which are a potential tumorigenic factor together with gastric pH were not measured. Secondly, enhanced bacterial overgrowth, another potential important mechanism for omeprazole’s effect on gastric carcinogenesis, could not be addressed in this study. Thirdly, due to the fact that we were not really wishing to robustly induce GC but just to supply an N-nitroso-compound, the non-mouse-specific gastric carcinogen MNNG was used. The induction of mostly fore-stomach cancers which comprised the most of the malignant cases would be potentially in parallel to cancers of distal esophagus and esophagogastric junction, rather than stomach. Notably, more atypical hyperplasia cases occurred in the omeprazole treated group together with MNNG. Supplement of MNU in drinking water which is the most commonly used way to construct the mouse GC model with antral carcinomas robustly yielded was not used in this study, but might be able to better clarify the effect of omeprazole on antral cancer induction with a baseline quantity of antral GCs induced. Furthermore, pharmacologic studies using 3H- or C14-labeled omeprazole would better clarify whether it is taken up in the lysosomes of other cell types (*e.g.*, splenocytes and squamous cells) besides parietal cells of the stomach, so that whether the effects on the respective cells are due to the direct or indirect modulatory mechanisms by omeprazole could be determined.

Nevertheless, this translational research provides the laboratory and theory basis for the clinical rational drug usage, and potentially explains the increase of AEG in Asian countries, particularly in China [3]. The results suggest that the mouse mucosal pathological changes are positively correlated with omeprazole dose, and reversely with lysosomal enzymes. Notably, omeprazole application was associated was an increased rate of squamous cell carcinoma in the fore-stomach, but not of adenocarcinoma in the glandular stomach. The findings could propose the potential clinical hypothesis that long-term PPI application might increase the incidence of adenocarcinoma of the GEJ among people with a significant intake of pickled food. For patients with digestive symptoms and frequent intake of pickled food, surgical, endoscopic or interventional management might be a better choice, compared to acid suppression drug prescription. For those who do require long-term administration, a routine gastroscopic examination is highly recommended, and the high-salt intake habit might need to be abandoned.

In conclusion, long-term omeprazole application increases the incidence of carcinoma of fore-stomach at the presence of N-nitroso-compounds in mouse. Lysosomal enzymes were inhibited and some tumor-associated proteins were disregulated, which however warrant further exploration. For long-term PPI users, avoidance of pickled food intake and regular gastroscopy examination might be necessary to avoid tumorigenesis.

## MATERIALS AND METHODS

### Ethics

All procedures were approved by the Association of Laboratory Animal Care at Anhui Medical University and complied with the relevant ethical guidelines concerning care and use of laboratory animals. This paper was in accordance with the ARRIVE guidelines.

### Experimental animals and reagents

A total of 70 specific pathogen free-grade 4-week old male Kunming mice with weight between 18-20 g were provided by the Experimental Animal Center of Anhui Medical University (certificate no.: SCXK2005-001). Omeprazole was from New Era Pharmaceutical Co. Ltd. (Shandong, China). MNNG was purchased from Tokyo Chemical Industry (TCI, Japan). The enzyme linked immunosorbent assay (ELISA) kits of N-acetyl-β-D-glucosaminidase (NAG) and acid phosphatase (ACP) were both purchased from Biomics Lab Co. Ltd. (Beijing, China).

### Construction and grouping of model animals

The mice were kept in the animal center of Department of Pharmacology at Anhui Medical University, and fed under standard conditions at 24 °C in polypropylene cages with free intake of food and water and with a 12-h light/12-h dark cycle. Four mice were excluded. After 3-day acclimatization, they were randomly divided based on an in-house computer system into 6 groups (11 mice per group, Figure 1), which were treated daily with normal saline as control, low dose omeprazole (6 mg/kg), high dose omeprazole (30 mg/kg), MNNG (100 mg/L) plus normal saline gavage, MNNG plus low dose omeprazole, and MNNG plus high dose omeprazole, respectively, for 24 weeks continuously. Omeprazole was delivered 40 mg/100 mL through gavage once a day, and MNNG was supplied in drinking water protected from light in foil packs for free intake. The dose of daily omeprazole was based on the dose for long-term clinical administration in humans (adults, 0.57 mg/kg; children, 0.7 mg/kg) and the metabolic ratio of mouse versus human being [50], and the MNNG concentration was based on that reported by previous animal experiments [51, 52]. Each mouse was weighed once per week after fasting for 12 h without water deprivation, and was deprived of food but allowed free access to water 24 h before sacrifice for specimens.

### Measurement of mouse spleen weight index (SWI)

After the last gavage of the experimental agent, the mouse was weighed before sacrifice. The spleen was obtained shortly after cervical dislocation, washed in 4 °C normal saline to get rid of the remnant blood, placed on ice with surrounding adipose tissues removed, dried on filter paper, and then weighed using an analytical balance. The SWI was calculated using the formula:

$$\text{SWI}=\frac{Spleen\;weight\;\left(mg\right)}{Body\;weight\;\left(g\right)}.$$

### Measurement of ACP and NAG in mouse serum and tissue

The blood was taken from the retro-bulbar venous of the mouse after anesthetized by intraperitoneal injection with 2.5% pentobarbital (2 mL/kg). After centrifugation for 10 min, the supernatant was obtained for measurement. The ACP and NAG levels in mouse serum and tissue were measured using the ELISA method following the manufacturer’s instructions. Briefly, the standards of different concentrations were firstly added, followed by loading of the diluted samples (1:5). The wells were added with corresponding horseradish peroxidase-labeled antibodies, and the reaction wells were membrane-sealed, followed by incubation at 37 °C for 60 min. The supernatants were discarded, and the plates were dried on absorbent paper. The wells were added with washing liquid and incubated for 1 min before discarding the liquid and drying on paper, for 5 times. Then the substrates were added, followed by incubation at 37 °C in dark for 15 min. After adding the stop reagent, the absorbance value (OD) was determined at 450 nm. The standard curve was generated together with the corresponding equation, and the protein concentrations of the samples were then determined.

### Pathological examination of mouse stomach

The mouse abdominal cavity was opened soon after sacrifice, and the stomach was obtained followed by normal saline rinse, placement on ice, and removal of the peri-gastric adipose tissues, and was then slit along the greater curvature. The specimen was rinsed for 3 times using 4 °C normal saline to remove the remnant intra-gastric food before fixation, and the gastric mucosa was observed macroscopically with photos taken. For each mouse, two tissues of the gastric fundus, body, and antrum were obtained respectively, and then fixed in 10% neutral formaldehyde solution for 24 h. Then the specimens were gradient alcohol-dehydrated, xylene-vitrificated, wax-embedded, 4 μm-sliced, and then stained using the hemotoxylin-eosin (HE) method, before observed microscopically with pictures taken. For the HE staining, briefly, slices were xylene-dewaxed, gradient alcohol-washed, dyed in hematoxylin for 30 min followed by tap water wash, placed in 1% hydrochloric acid-ethanol for 5 s followed by wash, stained by 0.5% eosin for 2 min followed by wash, dehydrated in gradient ethanol, and then vitrificated by xylene. The gastric mucosal slices were reviewed by experienced pathologists in a double-blinded way. The images were analyzed using the JEOR 801D Morphological Image Analysis System (V. 6.0) created by University of Science and Technology of China.

### Western blotting

The protein was extracted from the gastric tissue using an extraction buffer. The protein concentrations were determined by the bicinchoninic acid assay (BCA) method using the BCA Protein Assay Kit (Beyotime Institute of Biotechnology, China). Total protein extracts were separated using sodium dodecyl sulfate polyacrylamide gel electrophoresis (SDS-PAGE) gels. After electrophoresis, the proteins were transferred to a polyvinylidene difluoride (PVDF) membrane (Bio-Rad, Hercules, CA, USA). After blocked in tris-buffered saline (containing 5% milk and 0.3% Tween 20 (TBST)) for 1 h, membranes were probed with monoclonal antibodies, rabbit anti-p21 antibody (Abcam, Cambrige, UK), and rabbit anti-mammalian target of rapamycin (mTOR) antibody (Affinity Biosciences, Cincinnati, USA). After incubation overnight, the membranes were washed with TBST and incubated with Goat anti-rabbit IgG (H + L) horseradish peroxidase (HRP; Affinity Biosciences, Cincinnati, USA). The blots were visualized by the SuperSignal enhanced chemiluminescence (ECL) detection system according to the manufacturer’s instructions (Bioshine ChemiQ 4600 fluorescence and chemiluminescence imaging system, Ouxiang Scientific Instrument Co. ltd, Shanghai, China). The relative band intensity was then quantified.

### Statistical analysis

This study was based on intention-to-treat analysis. Measurement data were presented as mean ± standard deviation/error. For quantitative data, the comparison between multiple groups was first conducted using the one-way analysis of variance (ANOVA) method, followed by the Student-Newman-Keuls *q* test for multiple comparison if appropriate, and the comparison between 2 groups was performed using Student’s *t* test; for qualitative data, the *χ*^*2*^ test or the Fisher’s exact test was applied. A difference was considered statistically significant with *P* < 0.05, and very significant with *P* < 0.01. All *P* values were 2-sided. The IBM SPSS Statistics 20.0 and Microsoft Excel 2013 software was used for data management.
